# Advances in research and current challenges in the treatment of advanced HER2-low breast cancer

**DOI:** 10.3389/fcell.2025.1451471

**Published:** 2025-03-19

**Authors:** Qiang Qin

**Affiliations:** Breast and Thyroid Surgery Department, Nanning Maternal and Child Health Hospital, Nanning, China

**Keywords:** breast cancer, HER2-low expression, T-DXd therapy, treatment, HER2-targeted therapies

## Abstract

Human epidermal growth factor receptor 2 (HER2)-low breast cancer is defined as breast cancer with an immunohistochemistry (IHC) score of 1+ or 2+ and *in situ* hybridisation (ISH)-negative. The traditional HER2 classification (negative or positive) has limitations, with only 15%–20% of the breast cancer population being positive and suitable for HER2-targeted therapy. The new clinical study, DESTINY-Breast04, shows that trastuzumab deruxtecan (T-DXd) has a significant effect on advanced HER2-low breast cancers, a classification that accounts for approximately half of the advanced breast cancer population. However, the detection methods and evaluation criteria for HER2-low breast cancer have not yet been standardised, and the toxicity and resistance mechanisms associated with T-DXd therapy are still unclear. This article focuses on these issues and describes the progress and challenges of T-DXd-related therapy in the treatment of advanced breast cancer patients with low HER2 expression.

## Introduction

**FIGURE 1:**
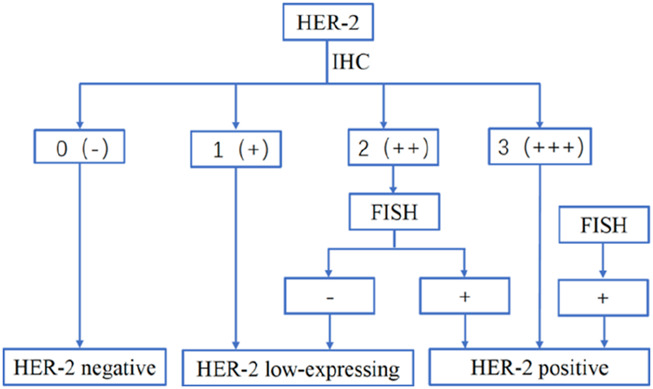
Classification of HER2-low breast cancer.

According to the World Health Organisation’s data for 2022, breast cancer has the second-highest globalincidence, following lung cancer (da Costa Nunes et al., 2024; Sung et al., 2021; Zheng et al., 2024). Human epidermal growth factor receptor 2 (HER2) is a well-recognised prognostic and predictive biomarker in breast cancer and plays an important role in the diagnosis, staging, and treatment of breast cancer (Cooke et al., 2001). HER2 is augmented in approximately one-fifth of patients, leading to a significant increase in the amount of HER2 protein on the cell surface and enhancing signal transduction through HER2 heterodimers, which results in cell proliferation and migration (Rubin and Yarden, 2001). Prior to the advent of anti-HER2 therapy, HER2-positive breast cancer had a higher risk of recurrence and shorter survival (Seshadri et al., 1993; Slamon et al., 1987). HER2-low breast cancer is predominantly found in hormone receptor (HR)-positive patients. Its pathological complete response (pCR) rate is significantly lower than that of HER2-0 breast cancer, particularly in HR-positive subgroups, whereas the difference is less evident in HR-negative patients. Moreover, the three-year disease-free survival (DFS) rate of HER2-low patients is higher than that of HER2-0 patients (Denkert et al., 2021). The PAM50 subtype of HER2-low breast cancer is mainly classified into Luminal A and Luminal B, whereas HER2-0 breast cancer is more commonly associated with the basal-like subtype. Genomic analysis indicates that HER2-low breast cancer exhibits slightly elevated ERBB2 gene expression levels. However, no significant differences are observed in terms of mutation frequency, copy number variation, or tumour mutation burden (TMB) between HER2-low and HER2-0 breast cancer (Schettini et al., 2021a). However, trastuzumab, pertuzumab, and other anti-HER2-targeted drugs have revolutionised the treatment and prognosis of patients with early and advanced HER2-positive breast cancer (Cameron et al., 2017; Early Breast Cancer Trialists' Collaborative g. Trastuzumab for early-stage, 2021; Piccart et al., 2021; Swain et al., 2020). HER2 expression can be detected using immunohistochemistry (IHC) and *in situ* hybridisation (ISH). It is defined as an HER2-IHC score of 3+ or 2+ with an HER2/CEP17 ratio above 2 and/or a HER2 copy number exceeding six signals/cell (Wolff et al., 2018). Approximately 50% of patients classified as “HER2-” exhibit low levels of HER2 expression; however, only about 15%-20% of tumors meet these criteria. (de Calbiac et al., 2022; Gampenrieder et al., 2021; Schettini et al., 2021b). These tumours are now referred to as HER2-low patients, who were IHC 1+/2+ without ISH amplification (Tarantino et al., 2023a). This group of patients makes up approximately half of the breast cancer population, indicating great potential for research in targeted therapies for HER2-low expression (Figure 1). Based on the results of the phase III trial DESTINY-Breast 04, a paradigm shift is expected in the treatment of HER2-negative advanced breast cancer. For example, trastuzumab deruxtecan (T-DXd) therapy provided a survival benefit in patients with advanced HER2-low breast cancer, suggesting that antibody–drug coupler (ADC) analogues may have a targeted killing effect on low HER2-expressing tumour cells (Modi et al., 2022) In the current study, we present the study of ADCs for HER2-low expression in advanced breast cancer patients and describe the progress and challenges in the treatment of advanced breast cancer patients with low HER2 expression. HER2-low breast cancer demonstrates potential differences in immune microenvironment characteristics compared to HER2-0 breast cancer, suggesting distinct mechanisms of immune regulation and infiltration (Tarantino et al., 2023b).

### Targeted therapy for conventional HER2-positive breast cancer

Current protocols are still based on three biomarkers, namely, HER2, oestrogen receptor, and progesterone receptor, to develop personalised treatment plans for breast cancer patients (Gradishar et al., 2022). HER2 expression has always been classified in a bipolar manner, i.e., HER2-positive or HER2-negative. HER2-targeted therapies for the treatment of HER2-positive advanced breast cancer involve monoclonal antibodies such as trastuzumab and pertuzumab; ADCs such as trastuzumab emtansine (T-DM1) and T-DXd; and the small-molecule tyrosine kinase inhibitors for HER2 (Gradishar et al., 2022; Gennari et al., 2021). The use of these drugs has considerably enhanced the overall survival (OS) in HER2-positive advanced breast cancer patients (Gradishar et al., 2022). However, trastuzumab, pertuzumab, and T-DM1 did not show meaningful benefits in the low HER2-expressing breast cancer population (Burris et al., 2011; Chick et al., 2021; Fehrenbacher et al., 2020; Filho et al., 2021; Gianni et al., 2010; Yazaki et al., 2020). Therefore, until now, breast cancer patients who have a low-expressing level of HER2 have been categorised as HER2-negative in terms of treatment decisions and receive routine treatment based on the expression of hormone receptors and other targeted biomarkers (Schettini et al., 2021b).

### Advances in research on the treatment of advanced breast cancer with low HER2 expression

**FIGURE 2:**
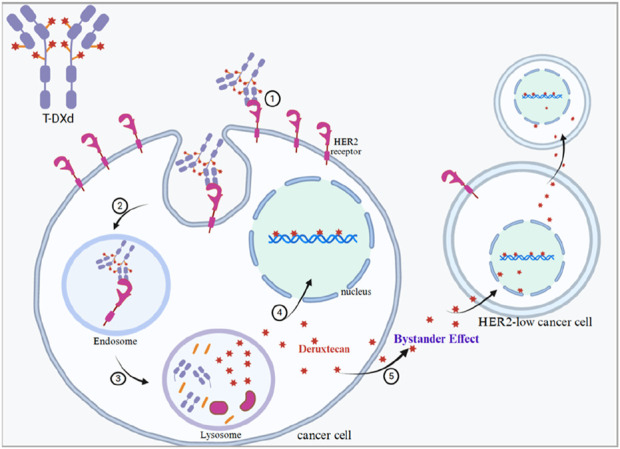
Mechanism of action of T-DXd. In the mechanism of action of antibody–drug couplers (ADCs) like T-DXd, internalisation and degradation are the key steps to achieve drug-specific killing of cancer cells. After binding to the HER2 receptor, the ADC enters the cell interior via endocytosis and is degraded in the special intracellular environment, releasing cytotoxic drug components (deruxtecan), which leads to the death of tumour cells. 1. Targeted binding of HER2 receptor. The antibody portion of T-DXd (i.e., trastuzumab) can specifically recognise and bind to the HER2 receptor on the surface of cancer cells. 2. Endocytosis process of ADCs. Upon binding of the HER2 receptor to T-DXd, cancer cells endocytose T-DXd along with the HER2 receptor through receptor-mediated endocytosis, forming endocytosed vesicles (endosome). 3. Entry of lysosomes for degradation. The endocytosed vesicles gradually fuse with the lysosomes within the cell. In the lysosome, the antibody portion of T-DXd and the drug linker attached to it are degraded by lysosomal enzymes, releasing the highly potent cytotoxic drug (deruxtecan) from T-DXd. 4. Release and action of cytotoxic drugs. After T-DXd is degraded in lysosomes, deruxtecan is released, which is a topoisomerase I inhibitor. The released deruxtecan acts directly on DNA topoisomerase I in cancer cells, interfering with DNA replication and transcription, triggering DNA breaks, and ultimately leading to cancer cell death. 5. Bystander effect. Deruxtecan is membrane-penetrating, and once released from within the targeted cancer cells, it can also spread to neighbouring cancer cells, even if these cells are low or even HER2-negative in HER2 expression.

Although HER2 overexpression has long been considered necessary for the efficacy of anti-HER2 targeted therapy, new evidence suggests a change in treatment philosophy with the development of new and more effective agents such as T-DXd (Ali and Graff, 2024; Tarantino et al., 2023c). This ADC treatment involves a humanised HER2 antibody linked via a cleavable junction to a membrane-permeable payload of topoisomerase I inhibitors. Once T-DXd binds to the HER2 protein, irrespective of the level of HER2 expression, it transports its toxic cargo (8:1 DAR) to cancer cells and adjacent cells through a bystander effect (Ogitani et al., 2016a; Ogitani et al., 2016b), which distinguishes T-DXd from conventional HER2-targeted therapies (Figure 2). In phase III clinical trial DESTINY-Breast03, T-DXd exhibited a significant improvement in OS compared to T-DM1, demonstrating that ADC therapeutic technology has a significant effect (Hurvitz et al., 2023). To better understand the survival benefit of T-DXd in patients with low HER2--expressing advanced breast cancer, the DESTINY-Breast04 phase III trial was conducted. The DESTINY-Breast04 clinical trial included 557 patients with hormone receptor (HR)-positive or HR-negative, low HER2-expressing unresectable and/or metastatic breast cancer who had received 1–2 lines of prior chemotherapy, and the patients were randomised to either the T-DXd group or the physician’s choice of the chemotherapy group (in a ratio of 2:1). The results of the DESTINY-Breast04 phase III clinical trial showed that in the total population, the OS of the T-DXd group and the chemotherapy group was 22.9 months and 16.8 months, respectively; in the HR-positive cohort, the OS of the T-DXd group was 23.9 months, compared with 17.6 months in the physician’s choice of the chemotherapy group; and in the HR-negative cohort, the median OS rates of the two groups were 18.2 months and 8.3 months, respectively (Modi et al., 2022). It is noteworthy that patients with advanced HER2-low breast cancer have traditionally been categorized under triple-negative breast cancer (characterized by the absence of estrogen receptor, progesterone receptor, and HER2 expression). Due to the lack of hormone receptor expression, these patients have limited efficacy from second-line and third-line chemotherapy and immunotherapy, poorer prognosis, and lack effective endocrine treatment options (Dri et al., 2024; Liu et al., 2023) In contrast, the results of DESTINY-Breast04 phase III clinical trial showed a statistically different and clinically significant benefit in OS, regardless of the HR status, which presents a new treatment option for the population of advanced recurrent triple-negative breast cancer. In addition, a subgroup analysis of brain metastases confirmed that T-DXd provides excellent intracranial outcomes in patients with low HER2--expressing advanced metastatic breast cancer (Modi et al., 2022). DESTINY-Breast04 is the first phase III trial to achieve positive results in patients with low HER2 expression, a breakthrough that means T-DXd may bring a new treatment paradigm to nearly half of the advanced breast cancer population. The DESTINY-Breast06 trial (NCT04494425), which is currently under investigation, is further exploring the efficacy and safety of T-DXd in the ultra-low expression of HER2 (IHC> 0 but <1+) breast cancer patients. Patients were assigned in a 1:1 ratio and randomised to receive either the T-DXd group or the chemotherapy treatment of physician’s choice (TPC). The primary study endpoint was progression-free survival (PFS) in the HER2-low-expressing population; the secondary study endpoints were OS and PFS in the HER2-overexpressing and ultra-low-overexpressing breast cancer populations. As of 18 March 2024, 866 patients (HER2-low, 713; HER2-ultralow, 153) were randomised; T-DXd significantly improved PFS in those with low HER2 compared with TPC [HR: 0.62 (95% CI: 0.51, 0.74), P < 0.0001; median 13.2 months vs 8.1 months]. HER2-ultralow results were in line with those of HER2-low (Curigliano et al., 2024). The clinical benefits demonstrated by T-DXd in the treatment of advanced breast cancer in later lines have not been matched by any other drugs. The DESTINY-Breast series of studies continue to confirm that current breast cancer systemic therapies are breaking traditional indications and exploring broader clinical applications through innovative treatment combinations. It is believed that T-DXd will bring more breakthroughs in breast cancer treatment in the future.

## Challenges in the study of HER2-low-expressing breast cancer

### Limitations and future challenges in treating HER2-low breast cancer

**FIGURE 3:**
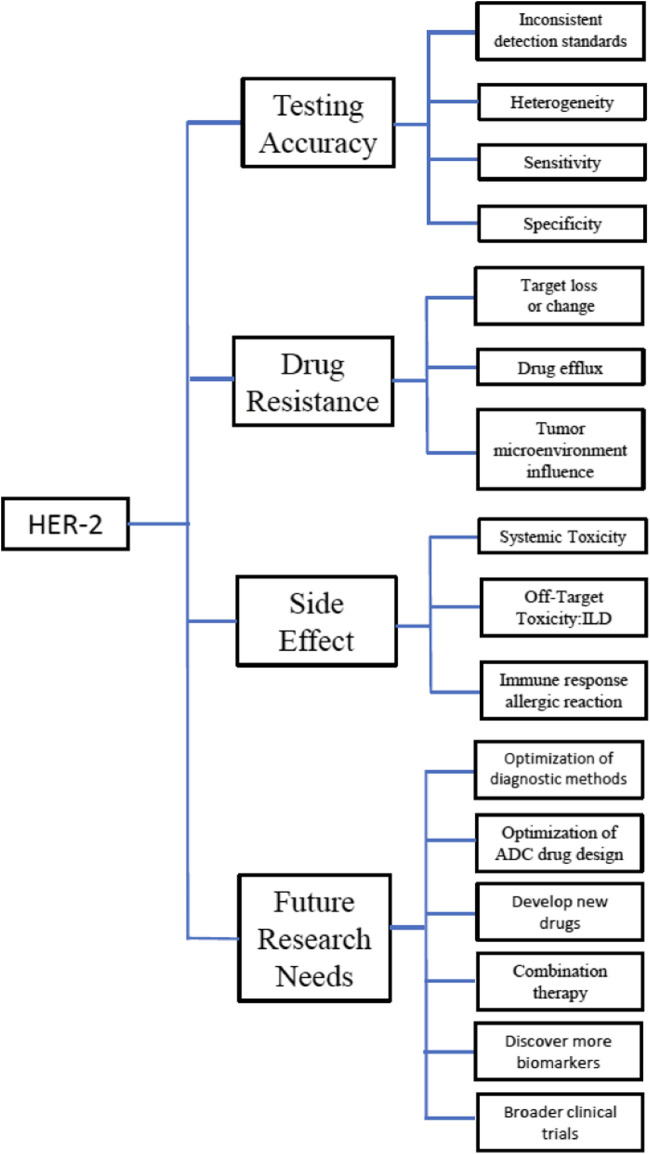
HER2low expression breast cancer. 1. Testing accuracy. Inconsistent detection standards: Low HER2 expression is usually defined as an IHC result of 1+ or 2+ without HER2 gene amplification, but IHC testing is subject to subjective judgement errors. For the interpretation of borderline values (e.g., between IHC 1+ and 2+), the level of training and experience of the technician can significantly affect the reliability of the results. In addition, variability across laboratories and equipment can introduce bias in results. Heterogeneity: HER2 expression within breast cancer tumours may be heterogeneous, i.e., HER2 expression levels may be inconsistent in different regions. This makes the detection of a single tissue section insufficient to accurately reflect the HER2 status of the whole tumour. Sensitivity and specificity: The diagnostic accuracy of HER2 low-expression may be limited by the method of detection. Although IHC and FISH are commonly used assays, the sensitivity and specificity of the assays may be lower in cases of low expression, leading to false-negative or false-positive results. Need for standardisation: A uniform clinical standard for low HER2 expression has not been fully established. Standardised and automated assays need to be explored to reduce human error and inter-laboratory variation. 2. Drug resistance. Target loss or change: Cancer cells may reduce the target binding of ADCs by decreasing HER2 expression, producing mutations, or adjusting receptor structure. In addition, after multiple rounds of treatment, some HER2-low-expressing cancer cells may further reduce HER2 expression or lose HER2 altogether, rendering ADCs less targeted. Drug efflux: The efficacy of ADCs relies on the internalisation of the drug by cancer cells and the release of toxic substances to kill the cells. However, cancer cells may alter the internalisation mechanisms of ADCs or change the metabolic and efflux pathways of the drug, thereby reducing the effects of ADCs. These mechanisms include the possibility that cancer cells may increase toxin efflux by overexpressing the upregulation of drug efflux pumps such as P-glycoprotein. Tumour microenvironment influence: Components of the tumour microenvironment, such as mesenchymal stromal cells, immune cells, and stromal components, may shield or reduce the effects of ADCs to some extent. For example, an increase in stromal components may prevent effective delivery of ADCs or activate local immunosuppression, reducing the overall efficacy of the drug. 3. Side effects. (1) Systemic toxicity. Systemic toxicity is a toxic response to a drug in the body that affects multiple organ systems, usually related to the nature of the drug itself, the dose, and the distribution of the drug in the body; cytotoxic drugs in ADCs not only act within tumour cells but may also enter the blood circulation and affect normal, rapidly dividing cells (e.g., bone marrow, gastrointestinal tract, and hair follicles). Damage to these cells causes a systemic toxic response. Systemic toxicity is not dependent on the targeting of ADCs; it is more of a general side effect of the drug. Common: bone marrow suppression: reduction in white blood cells, red blood cells, and platelets; gastrointestinal reactions: nausea, vomiting, loss of appetite, and diarrhoea. (2) Immune response allergic reaction. Acute allergic reaction: Some patients may experience an allergic reaction to the first administration of ADCs, which is related to the antibody component or toxin carrier. Symptoms include skin rash, shortness of breath, and increased heart rate, which may lead to anaphylaxis in severe cases. Antidrug antibody formation: ADCs are formed by coupling monoclonal antibodies and cytotoxic drugs through chemical linkage. Prolonged use of ADCs may induce the body to produce anti-drug antibodies (ADAs), which reduce the efficacy of the drug or increase the risk of allergic reactions. This antibody response not only reduces the efficacy of ADCs but may also trigger other adverse reactions in the immune system. (3) Off-target toxicity. HER2 is not only expressed in cancer cells but also at low levels on many normal cells (e.g., liver, kidney, and heart cells). Although the expression of HER2 in normal cells is much lower than that in cancer cells, ADCs may still recognise and bind to HER2 on these normal cells. Once ADCs bind to and internalise HER2 on normal cells, the toxic drugs they carry are released in these normal cells, leading to cellular damage or even death. Non-specific release of toxic components may trigger functional impairment of normal tissues or organs. Liver injury: ADCs may mistakenly target HER2-low-expressing cells in the liver, triggering liver injury. As a major organ for drug metabolism and detoxification, the liver is more susceptible to off-target toxicity of ADCs. Renal injury: Low levels of HER2 expression in the kidney may also contribute to the release of ADC toxins. The kidney may be affected by off-target toxicity during drug excretion. Pulmonary toxicity: Some ADCs may trigger interstitial lung lesions. Cardiotoxicity: some cytotoxic drugs are cardiotoxic and may cause damage to the heart.

There are limitations and future challenges in treating HER2-low breast cancer, including assay standardisation, treatment-related toxicities, and emerging challenges in drug resistance (Figure 3).

### Problems with the accuracy of HER2-low-expression assays

Despite the favourable outcomes of the DESTINY-Breast04 trial, several issues remain in the diagnosis and treatment of patients with advanced HER2-low-expression breast cancer. The most critical issue is the accuracyof methods and assessment tools for detecting HER2, which directly affects patient outcomes (Fernandez et al., 2022). DESTINY-Breast04 adopted the VENTANA anti-HER2/neu (4B5) assay to classify HER2 expression based on the current criteria (Wolff et al., 2018; Modi et al., 2022). However, no standard assay is currently available to assess 1+ and 2+/ISH-disease (Modi et al., 2020a). In addition, IHC results are determined by factors such as HER2 kinetics, tumour heterogeneity, and instrumentation (Curigliano et al., 2024; Modi et al., 2020a). Furthermore, the distinction between the HER2 ultra-low expression range (0 and 1+) and differences in scoring consistency remain inconsistent and require further standardisation (Curigliano et al., 2024; Fernandez et al., 2022). New quantitative methods are also currently being explored by researchers, such as omics and AI analyses, and clinical trials have identified detection criteria for T-DXd (Hicks et al., 2018; Moutafi et al., 2022; Tarantino et al., 2020; Wu et al., 2023; Zoppoli et al., 2017). However, pathologists should be aware that, regardless of the HR status, the assessment of identified HER2 levels in patients with advanced breast cancer is clinically valid (Doi et al., 2017).

One of the critical challenges in diagnosing HER2-low-expression breast cancer lies in the inconsistency of current scoring methods, particularly in distinguishing between HER2 ultra-low (0 and 1+) levels. This variability is influenced by several factors, including the subjective interpretation of IHC results, tumour heterogeneity, and differences in assay platforms. Such inconsistencies can lead to the misclassification of the HER2 status, thereby affecting treatment eligibility and patient outcomes. Ongoing research is exploring advanced scoring techniques, such as digital pathology, quantitative PCR-based assays, and artificial intelligence-driven image analysis, which could help provide a more consistent and standardised assessment of HER2 levels. Continued efforts towards international standardisation, led by organisations like ASCO and CAP, are essential to improving diagnostic accuracy and ensuring appropriate treatment selection for patients with HER2-low- and ultra-low-expression breast cancer.”

### Treatment-related toxicity: interstitial lung disease

Although clinical symptoms and imaging appearances vary, interstitial lung disease (ILD) usually presents with lung inflammation and/or fibrosis, which is associated with many anticancer therapies. The diagnosis of ILD associated with T-DXd is challenging because it requires not only imaging, laboratory, and functional evidence but also the exclusion of infection (Cortes et al., 2022; Modi et al., 2020b; Powell et al., 2022; Yin et al., 2021). In the T-DXd arm of the DESTINY-Breast04 trial, 12.1% of patients developed ILD or pneumonia, a prevalence broadly consistent with previous analyses; however, three patients experienced a fatal event (Modi et al., 2022). Within the DESTINY-Breast06 trial, drug-related adverse events of grade (Gr) ≥3 occurred in 40.6% of patients (T-DXd) vs 31.4% of patients (TPC). A total of 49 patients (11.3%; 0.7% grade 3/4 and 0.7% grade 5) treated with T-DXd developed adjudicated interstitial lung disease/pneumonia compared to one patient treated with TPC (0.2% grade 2). The molecular mechanism by which ADC therapy causes ILD and pneumonia is still being investigated; it is possible that epithelial cells also have HER2 expression, and therefore, T-DXd could act on lung tissue and cause an adverse reaction. However, the existence of this mechanism is currently inconclusive (Press et al., 1990). Some animal studies have shown that T-DXd is localised to alveolar macrophages, thus suggesting an off-target mechanism, and cytotoxic lung injury is now considered the cause of T-DXd-associated ILD (Swain et al., 2022). However, until additional research data are available to clarify the pathogenesis of ILD, clinicians need to pay attention to treatment and patient characteristics that may increase the risk of ILD and pneumonia, such as drug dose, baseline oxygen saturation, moderate/severe renal impairment, and certain pulmonary comorbidities, and active monitoring can successfully identify these adverse effects (Wu et al., 2023; Yin et al., 2021). Active treatment should include early application of glucocorticoids and interruption of therapy (Powell et al., 2022; Swain et al., 2022). Whether re-treat with ADC after interruption is appropriateremains unclear, and re-treatment is currently only recommended for patients with low-grade ILD or pneumonia as there is no evidence supporting its use in patients with grade 2 or higher adverse events. More data from future clinical studies are needed to further improve patient safety (Rugo et al., 2022).

### Effect of T-DXd on HR-negative breast cancer

HER2-overexpressing breast cancer treatment might be dependent on HR expression. Although comparable objective remission rates were observed in T-DXd-treated HR-positive and HR-negative patients, there was a clear distinction in the OS of the two HR groups (Modi et al., 2022). However, this result is debatable due to the relatively small number of HR-negative patients in DESTINY-Breast04, which accounted for only 11.3% of the total patients. This proportion is certainly consistent with the fact that patients with HER2-low-expression early-stage breast cancer would have been mostly HR-positive. Therefore, more HR-negative patients need to be enrolled in this aspect of the T-DXd study.

Zhimin Shao et al. showed that in the Chinese triple-negative breast cancer population, HR-negative low HER2-expressing breast cancers were mainly dominated by non-basal-like disease, the proportion of which was significantly higher than that of the HR-negative HER2-0 patients. Regarding the molecular characteristics of non-basal-like tumours, the PI3K-AKT pathway was upregulated, and the frequency of PIK3CA mutation was higher than that of the HER2-0-expressing basal-like tumours (Dai et al., 2023). This suggests that HR-negative, low HER2-expressing breast cancers have distinct pathological types and molecular features from the HR-positive and HER2-0 populations, which may have a differential response to the treatment of T-DXd. However, more evidence is required; to date, T-DXd remains an important and effective therapeutic option for these patients.

### Drug resistance to ADC therapy

The mechanisms through which resistance occurs when receiving ADC therapy are still being investigated, and several theories have been put forward as to the causes of resistance, including reduced antigen levels/presentation, defects in drug internalisation and transport, abnormalities in lysosomes, and many changes in the cell proliferation and apoptotic signalling (Collins et al., 2019; Perez-Garcia et al., 2023), the alteration of which may, however, be only attributed to a specific type of structures, linkers, and cargos of ADC (Collins et al., 2019). Clinical research data confirm that patients who fail treatment with trastuzumab combined with paclitaxel can still benefit from T-DM1 therapy, suggesting that the antitumor activity of T-DM1 may have a unique mechanism of action, independent of previous anti-HER2 treatments (Amiri-Kordestani et al., 2014). However, additional research data still need to be conducted to confirm this.

The HER2-low-expression phenotype accounts for approximately half of the population with advanced breast cancer. The results of DESTINY-Breast04 highlight HER2 low-expression as an applicable target for advanced breast cancer patients, which is a departure from previous treatment concepts. The findings from the T-DXd clinical trial are transforming current clinical practice, changing the categorisation of advanced breast cancer and the global approach to the treatment of HER2-low-expression breast cancer. However, more research is still required to understand the underlying mechanisms of adverse ILD and drug resistance and provide safer and more effective treatments for clinical practice (Table 1). In the future, more detailed molecular typing can be incorporated into the T-DXd studies to develop more precise and individualised treatment plans and study the responsiveness of different molecular subtypes to T-DXd in HER2-low-expression breast cancer. In addition, the development and standardisation of testing methods and evaluation criteria for HER2-low-expression breast cancer will be conducive to the clinical application of T-DXd in HER2-low-expression advanced breast cancer.

**TABLE 1:** HER2-low breast cancer past and present treatments.

Treatment category	Specific treatment	Patient population	HER2 status	Outcome/effectiveness
Traditional chemotherapy	Anthracyclines and taxanes	Advanced BC	HER2-low, HER2-negative	Modest response, limited options
Hormone therapy	Tamoxifen and AI	HR-positive HER2-low	HER2-low, HR+	Beneficial for HR + subset
HER2-targeted ADCs	T-DXd and T-DM1	HER2-low	HER2-low, mixed HR+/−	Significant OS improvement for T-DXd
Emerging therapies	T-DXd + [combination agents]	HER2-low, ultra-low	HER2 ultra-low	Ongoing trials show promise

## Future research needs

Optimisation of diagnostic methods: Further optimisation and standardisation of accurate diagnostic methods for HER2 low-expression are still needed.

Optimisation of ADC drug design: Current ADC structures may not be best suited for HER2-low-expression breast cancer. Optimisation of next-generation ADCs in terms of drug toxicity, antibody specificity, and delivery efficiency could improve therapeutic efficacy, for example, by improving antibody binding or adapting toxin types to enhance the killing of low-expressing HER2 cells.

Develop new drugs: There is still a large gap in the treatment of patients with HER2-low-expressing breast cancer, and although T-DXd has shown good efficacy, there is still a need to explore more new targeted drugs and ADCs. These drugs should be able to effectively combat tumours with low HER2 expression or HER2 heterogeneity and improve the response rate of treatment.

Combination therapy: Considering the complexity of HER2-low-expressing breast cancers, future research may focus on multi-targeted drug combinations to overcome drug resistance and improve treatment efficacy. For example, combining anti-HER2 therapy with immune checkpoint inhibitors, chemotherapy, and hormone therapy may help enhance efficacy.

Discover more biomarkers: To better predict the treatment response in patients with HER2-low-expressing breast cancer, future research needs to identify new biomarkers.

Broader clinical trials: Large-scale, multicentre clinical trials can provide stronger evidence to evaluate the efficacy and safety of ADCs in HER2-low expression breast cancer. Meanwhile, efficacy and adverse effect studies based on real-world data can further validate the efficacy of ADCs in different populations and clinical settings.

## Summary

The diagnosis and treatment of HER2-low-expressing breast cancer face multiple challenges, including uncertainty in detection methods, the development of treatment resistance, the risk of adverse drug reactions, and the need for further research. To better address these challenges, future studies should focus on optimising detection techniques, developingnovel targeted therapeutics, and exploring the potential of drug combination therapy. In addition, biomarker discovery and the application of individualised treatment strategies will provide more effective therapeutic options for patients.
